# Assessing Organ Doses from Paediatric CT Scans—A Novel Approach for an Epidemiology Study (the EPI-CT Study) ^†^

**DOI:** 10.3390/ijerph10020717

**Published:** 2013-02-18

**Authors:** Isabelle Thierry-Chef, Jérémie Dabin, Eva G. Friberg, Johannes Hermen, Tore S. Istad, Andreas Jahnen, Lucian Krille, Choonsik Lee, Carlo Maccia, Arvid Nordenskjöld, Hilde M. Olerud, Kaddour Rani, Jean-Luc Rehel, Steven L. Simon, Lara Struelens, Ausrele Kesminiene

**Affiliations:** 1 International Agency for Research on Cancer, 150, Cours Albert Thomas, Lyon 69008, France; E-Mail: kesminienea@iarc.fr; 2 Belgian Nuclear Research Centre, SCK·CEN, Boeretang 200, Mol 2400, Belgium; E-Mails: jeremie.dabin@SCKCEN.BE (J.D.); lara.struelens@SCKCEN.BE (L.S.); 3 Norwegian Radiation Protection Authority, P.O. Box 55, Østerås 1332, Norway; E-Mails: Eva.Friberg@nrpa.no (E.G.F.); Tore.Istad@nrpa.no (T.S.I.); Hilde.Olerud@nrpa.no (H.M.O.); 4 Public Research Center Henri Tudor, 29, Avenue John F. Kennedy, 1855, Luxembourg; E-Mails: johannes.hermen@tudor.lu (J.H.); andreas.jahnen@tudor.lu (A.J.); kaddour.rani@tudor.lu (K.R.); 5 Institute of Medical Biostatistics, Epidemiology and Informatics, University Medical Center of the Johannes Gutenberg University Mainz, Obere-Zahlbacher-Str 69, Mainz 55131, Germany; E-Mail: krille1@imbei.uni-mainz.de; 6 Radiation Epidemiology Branch, Division of Cancer Epidemiology and Genetics National Cancer Institute, NIH, DHHS 6120 Executive Blvd. EPS Rockville, MD 20852, USA; E-Mails: leechoonsik@mail.nih.gov (C.L.); ssimon@mail.nih.gov (S.L.S.); 7 CAATS 43, Bd Maréchal Joffre, Bourg-La-Reine 92340, France; E-Mail: carlo-maccia@neuf.fr; 8 Clinical Epidemiology Unit, Department of Medicine, Karolinska Institute, Karolinska University Hospital, M9:01 SE-171, Stockholm 76, Sweden; E-Mail: arvid.nordenskjold@ki.se; 9 University in Oslo, Institute of Physics, P.O. Box 1072 Blindern, Oslo 0316, Norway; 10 Research Centre for Automatic Control, University of Lorraine, 34 cours Léopold, Nancy cedex 54052, France; 11 Radiation Protection and Nuclear Safety Institute, PRP-HOM/SER/UEM, BP 17, Fontenay-aux-Roses 92 262, France; E-Mail: Jean-luc.rehel@irsn.fr

**Keywords:** paediatric computed tomography, organ dose reconstruction, scanner settings, leukaemia, cancer risk

## Abstract

The increasing worldwide use of paediatric computed tomography (CT) has led to increasing concerns regarding the subsequent effects of exposure to radiation. In response to this concern, the international EPI-CT project was developed to study the risk of cancer in a large multi-country cohort. In radiation epidemiology, accurate estimates of organ-specific doses are essential. In EPI-CT, data collection is split into two time periods—before and after introduction of the Picture Archiving Communication System (PACS) introduced in the 1990s. Prior to PACS, only sparse information about scanner settings is available from radiology departments. Hence, a multi-level approach was developed to retrieve information from a questionnaire, surveys, scientific publications, and expert interviews. For the years after PACS was introduced, scanner settings will be extracted from Digital Imaging and Communications in Medicine (DICOM) headers, a protocol for storing medical imaging data. Radiation fields and X-ray interactions within the body will be simulated using phantoms of various ages and Monte-Carlo-based radiation transport calculations. Individual organ doses will be estimated for each child using an accepted calculation strategy, scanner settings, and the radiation transport calculations. Comprehensive analyses of missing and uncertain dosimetry data will be conducted to provide uncertainty distributions of doses.

## 1. Introduction

The use of computed tomography (CT) scanning in paediatric radiology departments has increased rapidly worldwide over the past two decades. CT is now a standard modality in assessing a variety of disorders in children as well as for cancer detection and surveillance, and evaluation of trauma and inflammation.

CT typically deliver doses that are substantially greater than those received from conventional radiographic examinations and the growing use of CT procedures in children and adolescents is, therefore, a topic of widespread concern [1,2,3,4,5,6]. Issues of concern unique to the paediatric population include increased radio-sensitivity of certain tissues, particularly in infancy, and a longer lifetime for radiation-related cancer to develop. Several studies of CT use in children have been initiated, including a recent study by Pearce *et al.* [7] which found a positive association between radiation doses from CT scans and increased risk of leukaemia and brain tumours [7].

To improve upon earlier conducted CT risk studies, the EPI-CT study (Epidemiology study to quantify risks for paediatric computerised tomography and to optimise doses [8]) was developed and formally began in 2010. In particular, EPI-CT aims to increase the statistical power, *i.e.*, the power to reject the hypothesis of no health effects should it be false, with the additional objective of refining the risks earlier estimated. Both goals are particularly important for epidemiologic studies concerned with public health issues. In order to increase statistical power in the EPI-CT study, a large cohort of children who underwent CT examinations in nine European countries (Belgium, Denmark France, Germany, Spain, Sweden, the Netherlands, Norway and the United Kingdom) is currently being assembled. The primary objectives of EPI-CT are to evaluate the radiation-related risk of leukaemia and solid cancers using refined individual organ dose estimates. The study data will also be used to describe patterns of use of CT over time and between countries, to develop methods to characterize the quality of CT images in relation to dose, and to provide recommendations for CT dose optimisation for paediatric patients in Europe. In parallel, biological markers of CT-irradiation effects will be tested as part of a pilot study to be performed on a small subsample of the cohort members.

In order to assess the risk of cancer (primarily leukaemia) as a function of radiation doses received from CT scans in childhood and adolescence, cumulative individual doses to specific relevant organs need to be estimated for all subjects in the study. In EPI-CT, organ- or tissue specific dose estimates will be derived for red bone marrow (for analyses of leukaemia risk), for the brain (for analyses of brain tumour risk) and colon (for analyses of risk of all cancers other than leukaemia). In the future, doses may also be needed for other organs/tissues of interest (including breast and thyroid) for the evaluation of the radiation related risk of other specific types of diseases.

A reliable assessment of the actual dose received by a patient who undergoes a CT examination requires both information on the specific make and model of CT machine, its inherent parameter settings, and information about the CT imaging protocol. During the feasibility phase of the project, named the CHILD-MED-RAD project, possibilities to retrospectively assess individual organ doses were evaluated and a methodology was proposed, as presented in the following section.

## 2. CT Dosimetry

CT imaging is based on the use of an X-ray beam as a source of radiation from which attenuation through the body is measured and a data set is formed based on multiple positions of the source as it rotates around the body. From the data, reconstruction algorithms produce tomographic “slices” of the human anatomy. The radiation dose at any point in the body from a CT examination is the sum of the radiation doses received from all positions of the tube that would have exposed that point. CT imaging technology has improved over the years since its inception and will likely continue to change and evolve in the future*.* Because of the range of years during which cohort members might have received CT scans (from the mid 1980s until now), it is necessary that we include a variety of models of CT scanners in order to capture the range of possible exposures. Organ dose estimation requires taking account of different types of CT scanners, different CT scan technologies, and details of the CT examinations and protocols.

In the past few years, numerous technological improvements have been implemented by manufacturers in order to reduce the level of patient’s exposure, considering in particular, the sensitivity of paediatric patients. The improvements include: shorter scanning time which time allows for sophisticated examinations on very young patients, new software programs designed for small size patients, more sensitive multi-detector systems and improved collimation, lower kV settings, tube current (mA) modulation, shorter beam geometry and filter shaping. Together with the improvements oriented towards reducing patient dose, manufacturers have also made substantial efforts to maintain the overall quality of the produced images (level of noise and/or level of contrast).

The reliability of estimated patient doses, therefore, strongly depends on:

1. Availability of information on factors such as age, body size, sex, examination type and CT scanner model;
2. Availability of manufacturer’s technical information which would allow appropriate modelling of X-ray beam quality used to produce CT scan images;
3. Knowledge of CT protocols (technical settings, number of scans, scanned body limits, *etc.*).

Access to the data described above will ensure that simulations can reliably estimate doses which we know to vary substantially from one medical centre to another, even for the same exam type. The present project follows a feasibility study (Child-Med-Rad) [9] which was conducted in 2008–2009 in the participating countries. As participating countries considered various dates of start for recruitment of retrospective cohorts (as early as 1984), a minimum set of input data that is required for a crude estimation of the dose was discussed. The set includes:

(1) patient’s sex and age, the name of radiology department;
(2) year of scan, scanner type (or at least scanner generation);
(3) body area scanned (possibility to be extracted from examination type);
(4) date of scan;
(5) number of scans performed.

During the feasibility study, radiology departments considered to be included in the study in each country were contacted and both-archived records and digital recording systems currently in use were checked to ensure that the minimum set of parameters will be accessible.

The information collected on the patients is a very important source of data for assessing radiology practices and analysing ways to optimise doses. A separate working group has therefore been set up to work on the optimization concept. The group will first analyse technology developments in the past decades focusing on the manufacturers algorithms used for dose reduction. Existing guidelines for optimization in paediatric CT procedures will be updated to redefine quality criteria for the paediatric images. An electronic audit tool to assess image quality on real images will be developed. In EPI-CT, the scoring of image quality will be performed in parallel to the “automatic” assessment of dose. A small group of radiologists will be involved in assessing image quality on a subset of images (focusing on head CT). The exercise will be performed with radiology departments representative of the hospitals involved in the cohort study and an intercomparison of scores will be conducted. In practice, the audit tool could also be used by radiologists for regular self-surveys. A new CT technical phantom to perform objective dosimetry and image quality assessment in paediatric CT will also be conceived, in agreement with the ICRU requirements [10].

Current CT dosimetry methods can be categorized into direct measurement methods and computer simulations of radiation transport in the human body. Direct measurement methods generally use small-size dosimeters placed inside or at the surface of physical phantoms. The strength of these methods is the direct measurement of a dose metric which is well characterized and understood because the conditions are standardized. However, there are a number of limitations, which make direct methods inappropriate for this study. Primarily, the limitations include impossibility of conducting experiments for all CT scanners, CT examinations, CT protocols, patient sizes, and the difficulty in converting the measurement to an organ dose for a specific patient.

Monte Carlo computer simulation methods using computer phantoms have been successfully used for organ dose estimation in CT and other types of medical imaging. Hermaphrodite mathematical phantoms of different body sizes, scaled from adult to baby, coupled with conversion coefficients derived by Monte Carlo simulation have been made available by dosimetry laboratories (for example the former National Radiological Protection Board (NRPB) (now Health Protection Agency, HPA) in UK and the German National Research Centre for Environment and Health (GSF)) for a variety of X-ray beam qualities commonly used in diagnostic radiology [11,12,13,14,15]. Since the EPI-CT study includes males and females from birth to 20 years of age, and because radiation dose greatly depends on patient size, we found it necessary to generate a CT organ dose database with phantoms with smaller age gaps (from newborn to adults) and with more realistic anatomy. A consensus has been reached within the dosimetry experts group that hybrid computational phantoms are the most appropriate dosimetric tool to assess the CT scan organ dose [9]. Hybrid phantoms are based on non-uniform rational B-spline (NURBS) and polygon mesh surfaces and offer anthropomorphic flexibility and anatomical realism. Researchers at the University of Florida and the U.S. National Cancer Institute have developed a series of hybrid phantoms representing the ICRP 89 Publication [16] reference newborn, the reference male and female phantoms of 1 year, 5 year, 10 year old children as well as 15 year- adolescent [17]. The recent advances in hybrid phantom technology have resulted in the potential to greatly expand the range of patient ages and body sizes considered, with the possibility to calculate radiation dose to individuals of any specific height and weight. In addition to the application to a wide range of patient ages for both genders, other improvements in CT dosimetry include: ability to estimate radiation dose to any specific organ/tissue (e.g., blood vessel or bone marrow by bone site), improved bone marrow dosimetry (e.g., heterogeneous skeletal tissue), application to a various types of CT scanners (from older models to scanners which will be used in future years in the study centres), different CT scan technologies (e.g., mA modulation), and various types of CT examinations.

## 3. Strategy for Dose Reconstruction

**Figure 1:**
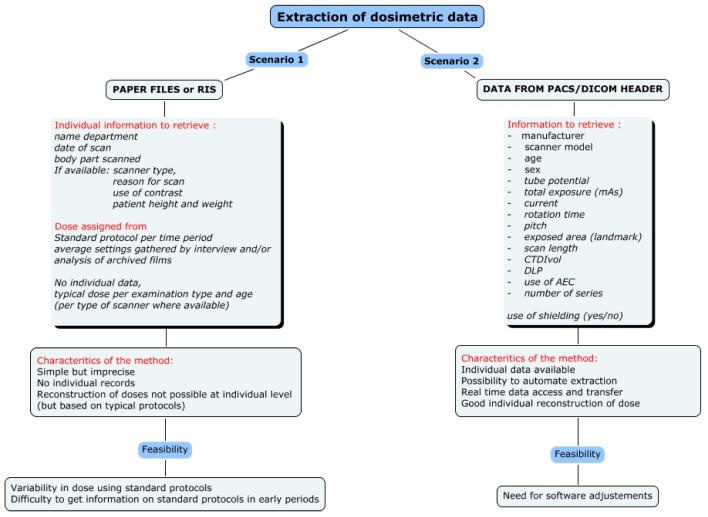
Scenarios for CT dose reconstruction.

In the context of EPI-CT, a strategy for dose reconstruction is needed that can accommodate varying degrees of information since the availability of the necessary information may vary between countries and among time periods. Two different dosimetric strategies will, therefore, be implemented, depending on the availability of information and the possibility to automatically extract that information (see Figure 1). In all participating countries, the Picture Archiving and Communication System (PACS), an information system used to acquire, store and retrieve medical images, was implemented in the late 1990’s to early 2000’s. The strategies, therefore, differ before and after the introduction of PACS.

### 3.1. Scenario 1: Radiological Records—Paper Files or RIS

For the time period prior to PACS, only limited information about scanner settings and technical parameters used for specific procedures can be obtained from paper files or from RIS (Radiology Information System) databases. A multi-level approach has been developed to retrieve information from a specifically developed questionnaire, published surveys, scientific publications, expert interviews and interpolations.

**Figure 2:**
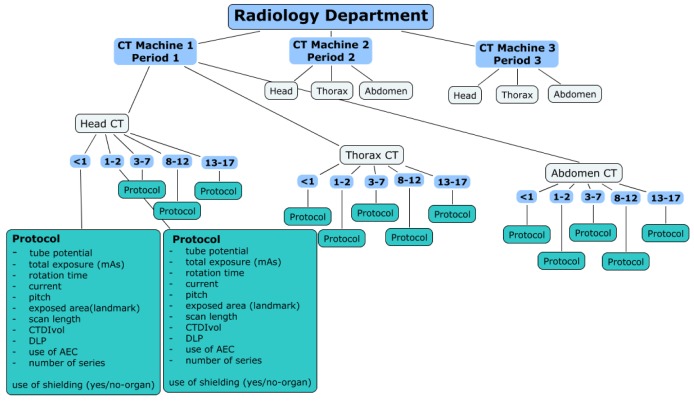
Structure of the questionnaire.

The questionnaire developed within EPI-CT is designed to document historical CT protocols for each patient age in each participating hospital, for a minimum of three anatomic zones (head, chest and abdomen). We believe that the main reason for protocols to have been modified over time was the change in CT machines (manufacturer and/or model), as shown in Figure 2.

Patients will be grouped by time period related to the use of different CT machines, examination types and by age, and organ doses will be estimated for each patient from typical protocols used at that time*.* It is anticipated that information on individual patients will be very limited. The patient data, most likely, will be limited to name/code of radiology department, date of scan, body part scanned, number of scans, sex and date of birth or age. Scanner type, reason for scan, use of contrast, height and weight may not be available. As noted, doses will be estimated based on typical protocols implemented in a specific hospital for the specific time period, procedure, age, and, where available, type of scanner. If available, archived images will be used to retrieve data on protocols.

### 3.2. Scenario 2: Radiological Records and Images Available—PACS or Archived Digital Images

**Figure 3:**
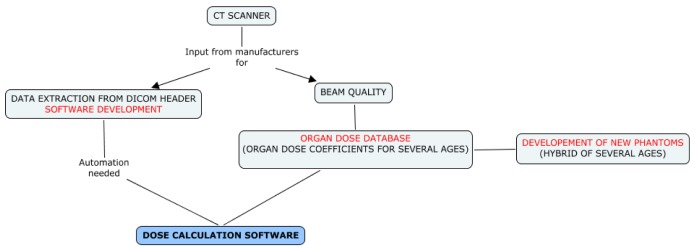
Dose reconstruction strategy in Scenario 2.

For the time period after the introduction of PACS, dosimetric data and technical scan parameters will be automatically extracted from DICOM (Digital Imaging and Communications in Medicine) headers of the images (Figure 3).

Dedicated software, such as PerMoS [18] which was adapted for a German pilot study of CT-related cancer risk in children [19], or Gladys [20], developed in parallel in Belgium, will be used within EPI-CT. In both cases, new modules have been developed specifically for the EPI-CT study to allow automatic recognition of the scanned body region (*i.e.*, identification of the organs in the primary field) using segmentation of the images.

Doses will be reconstructed individually from the examination settings recorded in the DICOM header including the number of scans (series) performed and the following parameters for each series: manufacturer and machine type, tube voltage, tube current, pitch and scan location (start and end of the scan). In addition, some information from manufacturers, or from measurements, on scanners including CTDIw values and mAs modulation (specification, algorithms) will be retrieved, when possible, to provide the most reliable dose estimates. This is particularly important for recent procedures that were individualised.

**Figure 4:**
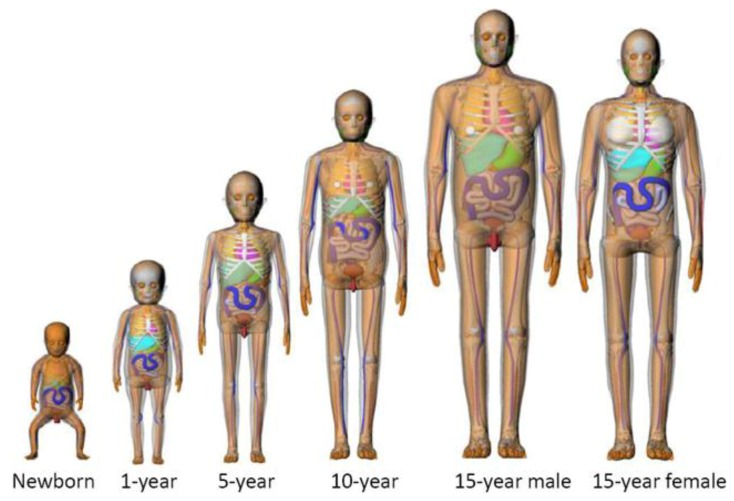
Family of hybrid phantoms [17].

Where available, height and weight information will be used to refine dose estimates by selecting a phantom that most resembles the stature of the patient. Some examples of the age-dependent phantoms are shown in Figure 4.

Using information on CT protocols, obtained either from the questionnaires (scenario 1) or from individual DICOM headers (scenario 2), radiation dose will be simulated by the Monte Carlo-based radiation transport method [21] to develop a set of organ doses which will be stored in a database whose entries will be available for selection based on each patient’s characteristics.

The possibility to include only patients with examinations performed in recent years was considered for the purpose of reducing the uncertainty that will unavoidably be present in dose estimates for earlier years. However, the size of the cohort ascertained retrospectively only through PACS, would be smaller—about 500,000–600,000 (assuming that the period of accrual ends in 2010). In this case, the follow up period would be quite short (~ 10 years, if analyses are planned in the next couple of years). The net effect would be to substantially reduce the statistical power needed to confirm risks of brain cancer and other solid tumours that might emerge in the short term. It was, therefore, concluded that patients from all time periods should be included so as to reach the necessary cohort size and that methods to characterize and account for uncertainties be developed.

## 4. Expected Results and Associated Uncertainties

As noted, organ doses will be derived based on data collected on various types of scanners used in nine countries in different time periods, together with information collected from standard examination protocols which are specific to scanner type, examination type and indication, hospital, and different ages/weights. It is useful to note that while historical information may not always be available, protocols were more standardized in the past and that typical protocols can be relied upon.

Accounting for missing parameter data is one of the main challenges, especially for earlier years (in the pre-PACS period), when we anticipate that only incomplete information on CT protocols in different hospitals will be available. A proposed approach to account for uncertainties in our dose estimates due to missing data is a simulation method [22,23,24] which maintains correlations of doses for persons within subgroups with similar attributes and simulates uncertain dose-model parameters values that could otherwise lead to biases if a single imputation strategy was used. We anticipate that the main parameters which are likely to be missing are: manufacturer and model of the CT machine, protocols for each age and examination type (mAs, kVp, pitch), and scanned area and body size of the patient. To allow for the range of possible true parameter values given that such data may be missing, each missing parameter necessary for dose calculation will be represented by a probability density function (PDF), representative of the state of knowledge about the conditions during the appropriate time period. The PDFs will be derived from all available sources of information for the corresponding time period (using, for example, data from similar hospitals in other countries, data from national surveys, *etc.*). The PDFs will likely be more precise for recent years when more information will be available and, thus, be narrower, *i.e.*, will be less uncertain. For each calculation of the cohort dose distribution, values of parameters will be selected from the appropriate PDFs while maintaining proper correlations between parameters. In simulations, emphasis will be put on estimating the dose for the entire cohort in order to maintain proper correlations among persons with similar (shared) attributes. The feasibility of applying this approach to our dataset is currently under investigation.

Our strategy for dose reconstruction is innovative for two major reasons. First, our assessment of organ doses will be performed for each participant individually, especially for recent years when characteristics of each examination can be retrieved. This is particularly important since in most of the cohort studies on medical diagnostic exposures, risk analyses are based on an estimate of the number of radiologic examinations received and not on individual organ doses. One of the best attempts to estimate CT doses to date was made in the UK cohort study in which doses were estimated based on typical CT machine settings as described in the UK National Surveys. A limitation of that study, however, was that no individual dose estimations were performed [25]. Secondly, since the level of detail available for dose reconstruction differs in different time periods, we believe that uncertainty in dose estimates, particularly when shared among members of a group (e.g., patients at a particular hospital), could be very important and, therefore, affect the reliability of the risk estimate. Hence, we are proposing to use alternative realizations of possibly true sets of doses for the cohort, each suitable for use in a dose-response as a surrogate of the true dose distribution as opposed to traditional single point estimates of dose for each person. The variability of dose for subjects with similar attributes will be represented within each realization of the cohort. The uncertainty of dose-related model parameters will be represented across all the realizations of the cohort.

Risk analysis with multiple realizations (*i.e.*, sets) of doses could be performed in one of several ways. The simple way of using the mean dose per person (from all the realizations) in the risk analysis may be adequate but is not yet confirmed. A more detailed approach would involve analysing all realizations of doses for each dose response, thereby fully accounting for inter-individual variability and uncertainty. The likelihood of each fit (or some other type of weight) could be used to obtain the most reliable estimate of risk and a confidence interval that reflects the state of knowledge.

## 5. Conclusions

Radio-sensitivity of the paediatric population is a topic of concern when considering exposure to ionizing radiation following CT imaging as radiation doses are substantially higher than from conventional radiographic procedures. Considerations unique to the paediatric population include increased radio-sensitivity of certain tissues, particularly in infancy, and a longer lifetime for radiation-related cancer to develop. A recent study [7] has shown increased risks of leukaemia and brain tumour at the level of dose delivered during CT scanning. That study is the first retrospective cohort study on the subject with personal information collected between 1985 and 2008 from the RIS database. Because no description of CT protocols and machines settings is available in RIS, dose reconstruction was, therefore, based on survey data only. In EPI-CT, which will include cancer incidence data from the UK study, we anticipate the possibility to improve individual organ dose assessment, especially for recent years, when technical parameters can be extracted automatically from the DICOM header of all images taken. It will, therefore, be possible to provide individual organ dose estimates for each CT examination performed on each of the cohort participants.

EPI-CT promises to be the most comprehensive assessment of CT-related risks due, in part, to the large cohort size and the resulting increase in statistical power. However, the improvements offered by EPI-CT will also be a result of improvements in dosimetry, in particular, by taking advantage of new strategies to collect scanner- and patient-specific data, use of the most realistic phantoms and radiation transport simulation, and dedicated attention to uncertainty analysis.

## References

[1] International Commission on Radiological Protection (ICRP) (2000). ICRP Publication 87—Managing Patient Dose in Computed Tomography; Report No. 30.

[2] Rehani M.M. (2000). Radiation doses in computed tomography. The increasing doses of radiation need to be controlled. BMJ.

[3] Brenner D.J., Elliston C.D., Hall E.J., Berdon W.E. (2001). Estimated risk of radiation-induced fatal cancer for pediatric CT. AJR Am. J. Roentgenol..

[4] Brenner D.J. (2002). Estimating cancer risks from pediatric CT: Going from the qualitative to the quantitative. Pediatr. Radiol..

[5] Committee to Assess Health Risks from Exposure to Low Levels of Ionizing Radiation, National Research Council (2006). US NRC Health Risks from Exposures to Low Levels of Ionizing Radiation; Report No. BEIR VII.

[6] Linet M.S., Slovis T.L., Miller D.L., Kleinerman R., Lee C., Rajaraman P., de Gonzalez A.B. (2012). Cancer risks associated with external radiation from diagnostic imaging procedures. CA Cancer J. Clin..

[7] Pearce M.S., Salotti J.A., Little M.P., McHugh K., Lee C., Kim K.P., Howe N.L., Ronckers C.M., Rajaraman P., Sir Craft A.W., Parker L., Berrington de González A. (2012). Radiation exposure from CT scans in childhood and subsequent risk of leukaemia and brain tumours: A retrospective cohort study. Lancet.

[8] Kesminiene A., Cardis E., Pearce M.S., Thierry-Chef I., Baatout S., Hauptmann M., Maccia C., Kaijser M. On Behalf of the Consortium. Epidemiological Study to Quantify Risks for Paediatric Computerized Tomography and to Optimise Doses (EPI-CT). www.cordis.europa.eu/projects/rcn/97571_en.html.

[9] International Agency for Research on Cancer (IARC) (2008). Prospective Cohort Studies of Children with Substantial Medical Diagnostic Exposure (Child-Med-Rad).

[10] International Commission on Radiation Units and Measurements (ICRU) (2013). Image Quality and Patient Dose in Computed Tomography, 2013; No. RC 19.

[11] Cristy M. (1980). Mathematical Phantoms Representing Child of Various Ages for Use in Estimates of Internal Dose; Report No. NUREG/CR-1159, ORNL/NUREG/TM-367.

[12] Zankl M., Veit R., Williams G., Schneider K., Fendel H., Petoussi N., Drexler G. (1988). The construction of computer tomographic phantoms and their application in radiology and radiation protection. Radiat. Environ. Biophys..

[13] Zankl M., Veit R., Petoussi N., Mannweiler E., Wittmann A., Drexler G. (1994). Realistic computerized human phantoms. Adv. Space Res..

[14] Jones D.G., Shrimpton P.C. (1993). Normalised Organ Doses for X-ray Computed Tomography Calculated Using Monte Carlo Techniques; Report No. NRPB-SR250.

[15] Impact Scan: CTDosimetry Version 1.0.4. www.impactscan.org/ctdosimetry.htm.

[16] International Commission on Radiological Protection (ICRP) (2003). Basic Anatomical and Physiological Data for Use in Radiological Protection: Reference Values; ICRP Publication 89.

[17] Lee C., Lodwick D., Hurtado J., Pafundi D., Williams J.L., Bolch W.E. (2010). The UF family of reference hybrid phantoms for computational radiation dosimetry. Phys. Med. Biol..

[18] Jahnen A., Kohler S., Hermen J., Tack D., Back C. (2011). Automatic computed tomography patient dose calculation using DICOM header metadata. Radiat. Prot. Dosimetry.

[19] Krille L., Jahnen A., Mildenberger P., Schneider K., Weisser G., Zeeb H., Blettner M. (2011). Computed tomography in children: Multicenter cohort study design for the evaluation of cancer risk. Eur. J. Epidemiol..

[20] Jacobs J., Marchal G., Bosmans H. Automated Patient Dose Evaluation for Pediatric CT, Proceedings of the Scientific Assembly and Annual Meeting of the Radiological Society of North America.

[21] Lee C., Kim K.P., Long D.J., Bolch W.E. (2012). Organ doses for reference pediatric and adolescent patients undergoing computed tomography estimated by Monte Carlo simulation. Med. Phys..

[22] Hofer E. (2008). How to account for uncertainty due to measurement errors in an uncertainty analysis using Monte Carlo simulation. Health Phys..

[23] National Council on Radiation Protection and Measurements (NCRP) (2009). Radiation Dose Reconstruction: Principles and Practices; Report No. 163.

[24] Simon S.L. (2009). The NCI Schema for Incorporating Uncertainty into Estimates of Radiation Dose in a Study of Thyroid Disease in Kazakhstan.

[25] Kim K.P., Berrington de González A., Pearce M.S., Salotti J.A., Parker L., McHugh K., Craft A.W., Lee C. (2012). Development of a database of organ doses for paediatric and young adult CT scans in the United Kingdom. Radiat. Prot. Dosimetry.

